# Evolution of spinal evoked compound action potential thresholds, visual motor thresholds, and impedances in a rodent spared nerve injury model

**DOI:** 10.3389/fnins.2025.1577059

**Published:** 2025-06-30

**Authors:** David L. Cedeño, Ricardo Vallejo, David C. Platt, Joseph M. Williams, Leonid M. Litvak, David A. Dinsmoor, Małgorzata Siorek

**Affiliations:** ^1^SGX Medical LLC, Bloomington, IL, United States; ^2^Illinois Wesleyan University, Bloomington, IL, United States; ^3^MiVi, Barcelona, Spain; ^4^Medtronic plc, Minneapolis, MN, United States

**Keywords:** evoked potentials, motor threshold (MT), neuropathic pain (NP), rat model, ECAPS

## Abstract

**Introduction:**

The mechanisms of spinal cord stimulation (SCS) on neuropathic pain are commonly studied using the spared nerve injury (SNI) model, with stimulation amplitudes typically programed relative to the visual motor threshold (vMT). Recent work explored the relationship between vMTs and spinal evoked compound action potential thresholds (ECAPTs)—a sensed measure of neural activation—in SNI rodents to better translate towards clinical dosing. However, changes across chronic healing beyond two days and pain states is unknown.

**Methods:**

This study tracked ECAPs through a traditional SNI-SCS approach, where nine rats were implanted with an SCS lead to evaluate effects of acute healing (days 0 to 1), chronic healing (days 1 to 7), nerve injury (days 7 to 14), and continuous SCS (days 14 to 16) using differential target multiplexed programing (DTMP).

**Results:**

ECAPT:vMT ratios significantly increased on subsequent recordings from day 0 through day 14 (i.e., post-injury), but not between days 14 and 16 (after SCS), across anesthesia states, or SCS pulse widths. On average, ECAPT:vMT increased from 35 ± 2% (mean ± S.E.) on implantation day to 54 ± 1% on day 16.

**Discussion:**

Future studies may use this approach to further elucidate the effects of chronic pain and SCS on the spinal ECAP.

## Introduction

Many preclinical studies exploring the mechanism of action underlying spinal cord stimulation (SCS) therapy leverage the spared nerve injury (SNI) model (Decosterd and Woolf, 2000; Tilley et al., 2015). This model is well-known to induce the mechanical and thermal allodynia that enables the study of the behavioral and neurophysiological effects of SCS. While some SCS parameters such as rate and pulse width (PW) may be employed identically between humans and preclinical models, delivering an equivalent dose remains a challenge owing to anatomical differences (such as cerebrospinal fluid thickness) across species (Chakravarthy et al., 2020; Cedeño et al., 2023b). Preclinical SCS models have historically titrated stimulation amplitudes to a percentage—such as 90%—of the visual motor threshold (vMT) (Crosby et al., 2015); the vMT is the SCS amplitude for which a muscle contraction is first observed. In contrast, SCS amplitudes in humans are often programed relative to the patient’s perceptual threshold (Al-Kaisy et al., 2020). As such, a significant difference can exist between the extent of neural activation in the preclinical model versus clinical use.

One method to realize equivalent neural activation both clinically and preclinically is to program SCS amplitudes relative to the evoked compound action potential threshold (ECAPT). The ECAPT—a measure of the onset of synchronized neural activation in the dorsal columns—is closely related to the paresthesia threshold in humans (Pilitsis et al., 2021). Previous work has now assessed both ECAPTs and vMTs in rats to provide context around legacy preclinical research that used vMT alone. In initial investigations (Dietz et al., 2022; Cedeño et al., 2023b), healthy, nerve injury-naïve rats were used; more recent work (Versantvoort et al., 2024) included a SNI model up to 2 days after lead implantation. These reports suggest that vMTs occur at approximately 3.0 times greater amplitudes than ECAPTs, a ratio similar to values noted in observational studies of rodents where the sensory threshold occurred at 40–50% of the vMT (Shechter et al., 2013; Song et al., 2014; Cedeño et al., 2023a). Other work has provided additional mechanistic insight into SCS by investigating novel evoked synaptic activity potentials (ESAPs) (Sharma et al., 2023), as well as differentially unique phenomena with low- versus high-rate stimulation (Sagalajev et al., 2024), though these studies were using acute terminal experiments in injury-naïve animals.

While important in elucidating dosing of SCS in translational neuropathic pain models, the aforementioned electrophysiology work in SNI rats only explores ECAPs for up to 2 days post implantation (Dietz et al., 2022; Versantvoort et al., 2024). However, as the acute inflammatory response transition to chronic healing, changes in encapsulation impact impedances (Arle et al., 2014; Garcia-Sandoval et al., 2018; Straka et al., 2018) and thus would likely impact neural activation. Additional translational insights from preclinical models of neuropathic pain may be realized by extending the study duration, with the effects of continuous SCS assessed over multiple days for rats (Tilley et al., 2015; Cedeño et al., 2020, 2023a; Vallejo et al., 2020; Wang et al., 2022) or weeks including larger models such as sheep (Reddy et al., 2018; Cedeno et al., 2022). Moreover, such models should employ lead designs and materials, such as platinum-iridium (Pt-Ir) electrodes, similar to those used clinically; the electrode composition in some prior reports is unfortunately not reported (Dietz et al., 2022; Versantvoort et al., 2024). Assessing the maturation of the electrode-tissue interface and the change in neurophysiological responses over the entire acute and chronic post-surgical interval is an important dimension to this class of work (Salatino et al., 2017; Shepherd et al., 2018; Carnicer-Lombarte et al., 2021).

In this study, the spinal ECAPs of rats—chronically implanted with a cylindrical SCS lead incorporating conventional Pt-Ir electrodes—were evaluated at multiple time points across a 16-day interval. The ECAPs were in turn related to evoked muscle activity via vMTs. In addition, tissue impedances were characterized longitudinally to evaluate changes in the electrode-tissue interface due to healing. Changes in ECAPs were further explored after inducing the SNI model as well as after continuous SCS, following a common SNI-SCS timeline (Vallejo et al., 2020). For the continuous SCS stimulation from days 14 to 16, DTMP-SCS was delivered as it has been shown to have a larger decrease in mechanical hypersensitivity than low-rate stimulation or high-rate stimulation through modulation of neuron-glial interactions (Cedeño et al., 2020, 2023a; Vallejo et al., 2020).

This longitudinal study approach allowed each animal to serve as its own control prior to the SNI. This study was primarily a feasibility study. We hypothesized we would see changes in neural activation and impedance immediately post implantation, and possibly additional changes after SNI. We did not expect further changes after chronic SCS given prior preclinical experience with continuously applied DTMP-SCS (Cedeno et al., 2022; Cedeño et al., 2023b). Neural activation was characterized via ECAPTs, slope of growth curves, latency, and conduction velocity measurements, and the changes in ECAPTs were related to those of the vMTs. While only recordings on anesthetized animals were performed at the day of implantation, both recordings in awake and anesthetized animals were collected starting on day 1 and onward to investigate the interdependency of anesthesia, time, and chronic pain state.

## Materials and methods

### Subjects and leads

All procedures were approved by the Institutional Animal Care and Use Committee at Illinois Wesleyan University following NIH guidelines for the ethical use of animal subjects in research. The authors complied with the ARRIVE guidelines. Ten male Sprague-Dawley rats (Envigo, Indianapolis, IN) approximately 12 weeks old weighing in the 297–320 g range at the start of the experiment were used in this study. The sample size used was selected for consistency with prior work (Cedeño et al., 2023b). Before experimental use, animals were housed in pairs in a temperature and humidity-controlled environment with a 12-h light/dark cycle. Food and water were supplied *ad libitum*. After lead implant, each animal was housed individually to prevent other animals from disturbing the external portion of the lead. Environmental conditions were kept similar to pre-implant. Animals were monitored daily for adverse events related to experimental interventions. An on-call veterinarian was available to assist with any serious event, though no such event occurred in the course of the study. Whenever isoflurane anesthesia was delivered, the animal was placed on a hot plate and the temperature was monitored to prevent hypothermia. Immediately after isoflurane, animals were allowed to recover in an empty cage to prevent inhalation of bedding material before returning to their cages. Some practical aspects of the experiment, including placing the reference needle and restraining the animal in a tube to minimize movement artifacts, may have caused the animal some stress and/or minor acute pain in the awake condition. To minimize this, animals were continuously visually monitored and other experimental interventions (e.g., behavioral testing) was minimized on recording days.

**FIGURE 1:**
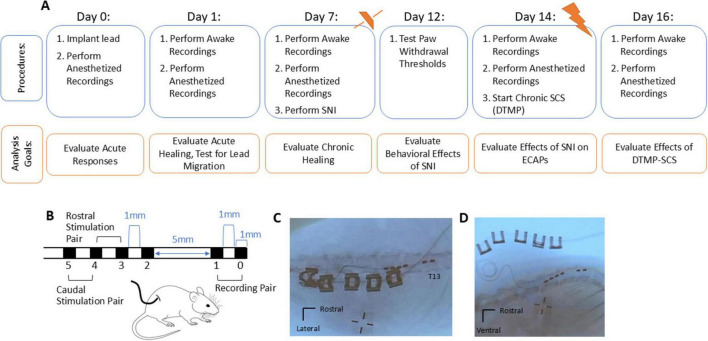
**(A)** A summary of the experimental design and timeline. ECAPs, vMTs, and impedances were recorded in the anesthetized rat during implantation (day 0). On subsequent days 1, 7, 14, and 16 after implantation, awake recordings were performed and then repeated when the animal was under isoflurane anesthesia. SNI was performed after recordings on day 7. The behavioral effect of SNI on paw withdrawal thresholds was evaluated on the injured limb and contralateral, non-injured limb on day 12, and the effect of the SNI pain model on ECAPs was evaluated on day 14. After recordings on day 14, continuous SCS via a DTMP paradigm was applied until day 16. **(B)** Mechanical dimensions and contact configuration of the implanted SCS lead, where the recording pair was placed most rostrally at T13. The SCS ring electrodes consisted of a 90–10% Pt-Ir alloy and each were 1 mm in length. The inter-contact spacing was 1 mm distance apart from the 5 mm distance to minimize the effect of electrical artifact on the propagating ECAP recorded on the recording pair e0/e1. ECAPs were recorded in response to 50 Hz stimulation for pulse width ranging from 50 to 200 μs applied with the rostral stimulation pair e3/e4 and (in separate recordings) with the caudal stimulation pair e4/e5. **(C,D)** The lead was placed epidurally spanning the T13-L2 vertebral segments as shown in post-mortem images of an exemplar rat.

A custom lead [cylindrical hexapolar lead (Oscor Inc., Palm Harbor, FL)] (Figure 1), comprised of 0.65-mm diameter ring electrodes (90–10% Pt-Ir alloy) each 1 mm in length, was implanted in each animal following a previously reported method (Tilley et al., 2015; Vallejo et al., 2020). The custom lead was designed to allow for DTMP-SCS at approximately L1-L3, targets similar to prior studies (Cedeño et al., 2020, 2023a; Vallejo et al., 2020) while also enabling the sensing of orthodromically propagating ECAPs in different recordings. The distal two recording electrodes (e0/e1) had an edge-to-edge spacing of 1 mm, followed by a gap of 5 mm between e1 and e2, with subsequent edge-to-edge spacing of 1 mm between the stimulating electrodes. The 5 mm spacing between recording and stimulating electrodes was selected to limit contamination of stimulation artifact on the ECAP recording (Chakravarthy et al., 2022).

### Experimental overview

Figure 1A shows a timeline for procedures and recordings with corresponding analysis goals. Briefly, stimulating electrical pulses (50 Hz) were delivered and both the orthodromically propagating ECAPs and vMTs were recorded. ECAPs were sensed on the recording pair of electrodes (e0/e1) in response to 50 Hz active recharge stimulation using a rostral stimulation pair (e3/e4) and, in separate recordings, using a caudal stimulation pair (e4/e5) (Figure 1B). On implantation day 0, only recordings under isoflurane anesthesia were performed; in subsequent days (days 1, 7, 14, and 16) recordings were first performed while awake and then under isoflurane anesthetized conditions. For each condition, ECAPs were sensed in response to electrical stimulation that was increased in 5 μA steps from 0 μA to just above when motor twitches were observed (vMT). Impedances were collected after recording ECAPs in each condition.

Recordings on day 1 post-implant were taken to evaluate neural activation and lead integrity, as well as check for any lead migration that may have occurred in the early post-surgical interval. In one animal, a notable change in ECAPs was observed at day 1, presumably due to lead migration. This animal was discontinued from the study, and its data excluded in the analysis. For the remaining 9 animals, healing over 1 week was evaluated via recordings on day 7 post-implant. The SNI was performed after recordings on day 7, and paw withdrawal thresholds (PWTs) for mechanical stimulus were collected on day 12 (i.e., 5 days post SNI) to confirm mechanical hypersensitivity associated with the SNI model. The effect of the SNI pain model on ECAPs was evaluated on day 14 post-implant (i.e., 7 days post-SNI). After recordings on day 14 post-implant, continuous DTMP-SCS was applied through day 16 post-implant. Recordings on day 16 assessed the carryover effect of continuous DTMP-SCS—if any—on neural activation.

After study completion, the animals were euthanized via carbon dioxide asphyxiation, and a subset of 5 cadavers were x-ray imaged to assess lead position (see Figures 1C,D for examples). While it was not possible to collect x-ray images for remaining animals in the study, the lead placement for the first 5 animals was confirmed to be within one vertebral body of the target T13, suggesting that this electrode array configuration and surgical technique enabled consistent placement. All data recorded were subsequently analyzed off-line. Further details are expanded below.

### Stimulating/recording and lead impedance measurement systems

A similar experimental setup as described previously (Cedeño et al., 2023b) was used to deliver stimulation and record ECAPs. Stimulation signals were sourced with a waveform generator (Model number 33511B, Keysight Technologies Inc., Santa Rosa, CA) and passed through a constant current stimulation isolator (Model number 2200, A-M Systems Inc., Sequim, WA). ECAPs were amplified (Model number D440, Biopac Inc., Goleta, CA), digitized (Model number MP160, Biopac Inc.) at a sampling rate of 40,000 samples/s, and stored on a laptop computer with recording software (AcqKnowledge v5.0, Biopac Inc.) that enabled streaming visualization of raw signals. The ECAPs were recorded differentially between the two most cranial electrodes (e0/e1) of the lead. Balanced, biphasic pulsed signals with a rate of 50 Hz, PW of 50, 100, 150, or 200 μs, and amplitudes from 0 μA to just above vMT in 5 μA steps were used to elicit ECAPs and vMTs. As each step was increased manually up to vMT, randomization of stimuli was not possible. For each animal, electrical stimuli were delivered on a rostral electrode pair (e3/e4) and, in separate recordings, on a caudal electrode pair (e4/e5). Impedances were recorded using an investigational device (ECHO-MDT, Medtronic plc) between all contacts pairs in response to square pulses at 100 Hz, 75 μA amplitude, and 80 μs PW. The influence of anesthesia state, contact configuration, and day after implantation on impedance values were evaluated.

### Lead implantation procedure and intraoperative ECAP monitoring (day 0)

The surgical procedure for implanting cylindrical leads in the dorsal epidural space of rats is described elsewhere (Vallejo et al., 2020). Briefly, animals were induced with 4% isoflurane anesthesia, and 3–3.5% isoflurane was maintained throughout the procedure. An incision was made over the lumbar spine, followed by a microlaminectory at L4 for anterograde implantation of the lead. The lead was introduced through the interlaminar space between L3 and L4 and positioned with the distal tip at T13. An amplifier reference needle (Model number 8227103, Medtronic plc) was placed subcutaneously near the base of the tail. ECAP growth curves, vMTs, and impedances were collected. Lead position was adjusted mediolaterally as needed to optimize ECAP recordings. The proximal cable of the lead was anchored internally in the musculature around the L5 spinous process, and the externalized cable secured to the skin using silk sutures and stainless steel clips. The external portion of the cable was looped within a homemade harness secured around the animal trunk. The reference needle was removed, anesthetics stopped, and the animal allowed to recover before returning to the colony.

### Chronic recordings procedure (day 1–16 post-implant)

Starting day 1 post implantation, recordings were performed first in awake, restrained animals. For these recordings, animals were placed in a tubular rodent holder for 250–500 g animals (Kent Scientific Torrington, CT, United States) with an amplifier reference needle placed subcutaneously near the base of the tail. The ECAPs and vMTs were recorded, followed by the impedance measurement, as described above. Restraining of the awake animal was used to limit variability in the spacing between the electrodes and the cord that might otherwise influence the repeatability of the measurements. After completing awake recordings, the animal was anesthetized as described above, and recordings were repeated to determine the effect, if any, of anesthesia.

### SNI procedure (day 7 post-implant)

The SNI procedure (Decosterd and Woolf, 2000; Vallejo et al., 2020) was performed following anesthetized recordings on day 7 post-implantation. This consisted of exposing, under isoflurane anesthesia, the sciatic nerve ipsilateral to motor stimulus at the level of its trifurcation to the sural, peroneal, and tibial branches, transecting and removing 1–2 mm of tissue from the tibial and common peroneal nerves while leaving the sural branch intact. To confirm induction of the SNI, the extent of mechanical sensitization was assessed 5 days after the procedure by measuring the PWTs of the injured and contralateral (non-injured) limbs. These were collected using standard von Frey filaments with an electronic esthesiometer (IITC Life Science, Woodland Hills, CA) (Martinov et al., 2023). We also analyzed the ratio of the two PWTs, where a ratio of 100% would indicate no impairment (i.e., failure of induction) as the limbs have a similar response, and low percentages would indicate high impairment (i.e., success of induction) as the affected limb would not tolerate the mechanical stimulus.

### Continuous SCS (day 14–16 post-implant)

Given that DTMP-SCS has shown to significantly decrease mechanical hypersensitivity after 48-hours of continuous stimulation in prior rat studies compared to low-rate stimulation or high-rate stimulation (Vallejo et al., 2020; Cedeño et al., 2023a), DTMP-SCS was continuously delivered after the recordings taken on day 14 though day 16 in a method similar to previous studies (Cedeño et al., 2023a). The electrode cables were connected to an external neurostimulator (ENS, 97725; Medtronic, Minneapolis MN) via a connector block fastened to a custom-made harness. The DTMP approach incorporates 50 Hz stimulation applied on contacts e2e3 with 150 μs PW, multiplexed with 3 × 300 Hz stimulation applied at contacts e4e5 with 50 μs PW. For consistency with prior reports, stimulation amplitudes were set to approximately 50% vMT at awake conditions on day 14 (range: 10–80 μA) (Cedeño et al., 2023a). Recordings were performed after SCS on day 16 to evaluate the effect of continuous SCS applied for 2 days.

### Data analysis

**FIGURE 2:**
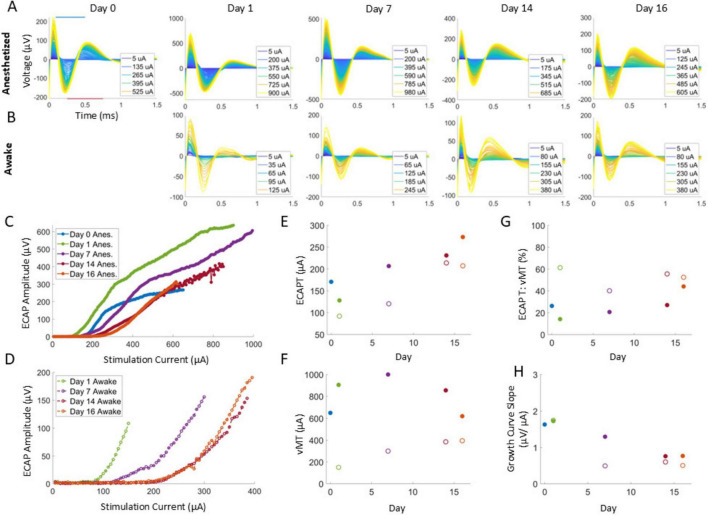
Example ECAPs for a representative animal in anesthetized and awake conditions **(A,B)** in response to 50 Hz, 50 μs PW stimuli. ECAP amplitudes were determined from the minimum and maximum within the N1 and P2 windows as indicated with the blue and red bars, respectively **(A,** Day 0 plot), to build growth curves **(C,D)**. For each condition at a given timepoint, the ECAPT **(E)** was calculated from the growth curve and compared to the collected vMT **(F)** via the ECAPT:vMT ratio **(G)** and the growth curve slope **(H)**. Legend in **(C,D)** are used for **(E–H)**.

All data was analyzed off-line using custom software in MATLAB 2020b (Mathworks, Inc., Natick, MA) similar to that used in a previous study (Cedeño et al., 2023b), with some modifications. Briefly, the stimulation artifact was first removed using an exponential modeling method, and then the resulting responses acquired at each stimulation amplitude step were averaged (Figure 2). The amplitude of the ECAP was calculated via the difference between the N1 and P2 features of the averaged polyphasic ECAPs. N1 was defined as the minimum voltage in the window between 0.09 and 0.5 ms after the end of a stimulation pulse, with P2 defined as the maximum voltage in the window between 0.25 and 0.75 ms after a stimulation pulse.

#### Determination of ECAPTs and vMTs

Growth curves were generated (see Figures 2C,D for examples) for each stimulation amplitude sweep by plotting the measured ECAP amplitudes versus the delivered stimulation current at a fixed PW. From each growth curve, the ECAPT was defined as the x-intercept resulting from fitting a regression line to the first three ECAP magnitudes above a threshold of 10 μV. If at least three points did not exceed 10 μV, then a threshold of 5 μV was used. Additional points were iteratively included for more accuracy if (1) the calculated slope did not decrease by more than 1% or (2) the x-intercept was negative or exceeded the max stimulation amplitude. In addition, the slope of the growth curve from the fit was also characterized.

The stimulation amplitude sweep continued until muscle motion was elicited by the stimulation current. The stimulation current at which this motion was observed was recorded as the vMT. For each growth curve, the ECAPT (Figure 2E for an example) and the concurrent vMT (Figure 2F for an example) were collected. The relationship between these were determined by calculating the ECAPT:vMT ratio (Figure 2G for an example).

Across all animals, 648 growth curves and corresponding ECAPTs were analyzed and visually assessed; 7 of which were manually corrected for accuracy. In addition, one growth curve was excluded from analysis due to partial data being saved for offline analysis. For the remaining 640 growth curves, the 95% Confidence Interval (CI) of individual ECAPTs was estimated using parametric bootstrap as detailed previously (Cedeño et al., 2023b). The slope of each of these 640 growth curves was calculated.

#### Determination of ECAP latencies and conduction velocities

The ECAP latency was calculated from recordings with the 50 μs PW stimulation delivered using electrode pairs e3/e4, and separately with pairs e4/e5. By utilizing the shortest PW, the potential for electrical artifact contamination on the ECAP recording was limited. First, the smallest average ECAP with at least a 20 μV amplitude in each recording was identified. In our recordings, 20 μV was well above the noise floor of the recording system but consistently below the point where the growth curves saturated as the stimulus amplitude increased. Then, latency was calculated by measuring the interval between the trailing edge of the stimulation pulse and the N1 feature of the 20 μV ECAP. Finally, we assessed both e3/e4 and e4/e5 latency differences as well as the conduction velocity (defined here as the separation between the proximal edges of e5 and e1 (12 mm) divided by the e5/e4 latency). Of the 162 growth curves, 5 did not have ECAPs larger than 20 μV and were not included in further analysis. Differences in latency between ECAPs elicited with both e3/e4 and e4/e5 SCS and collected after day 1 were made for the 68 of 72 growth curves (i.e., 4 differences were excluded because at least one of growth curves did not have the minimum size ECAP).

### Statistics

Summary statistics are reported as mean ± standard error unless otherwise stated. PWTs of injured and non-injured limbs were compared using a paired *t*-test with an alpha value of 0.05. For statistical comparisons of each of the impedances, growth curve slopes, ECAPTs, vMTs, ECAPT: vMT ratios, and latency, the longitudinal data were fit into a generalized linear mixed-effects (GLME) model where fixed effects were variables, and the random effect was the rat. Specifically, for impedances and latency, the fixed effects included the anesthesia state, the recording configuration, and the day post implantation. For ECAPTs, vMTs, ECAPT:vMT ratios, and growth curve slope, the fixed effects included the anesthesia state, the PW, and the day post implantation. All fixed and random effects were categorical variables for subsequent comparison tests. Additional information and summary variance statistics of each model can be found in the Supplementary Material. After fitting each model, estimated marginal means (emmeans function in MATLAB; Hartman, n.d.) were used for comparisons between specific variables (e.g., comparing between days or between pulse widths), with *p*-values reported after applying the Bonferroni correction for multiple comparisons.

## Results

Impedances, ECAPs, and vMTs were recorded across 16 days in nine rats that were chronically implanted with a hexapolar epidural lead. Following our traditional SNI-SCS approach, anesthetized recordings were performed from day 0 onward, along with awake recordings from day 1 onward, to evaluate effect of chronic healing (days 1–7), nerve injury (days 7–14), and continuous DTMP-SCS (days 14–16). For the 5 animals with post-mortem imaging, three had the most rostral contact on T13 with the lead spanning caudally to approximately the bottom of L2 or the top of L3. For the two remaining rats with imaging, the lead was placed caudally by a vertebral body (with the tip near L1).

### Validation of the SNI model

**FIGURE 3:**
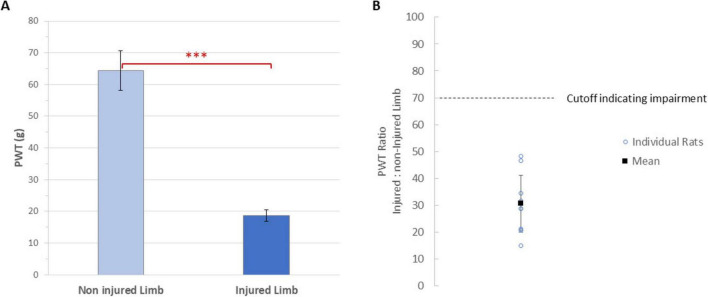
**(A)** Mean paw withdrawal thresholds of the injured and injured limbs of animals (*n* = 9). Error bars are standard errors. ^***^Indicates *P* < 0.0001. **(B)** The paw withdrawal threshold ratio for the injured to non-injured limb for each animal (circles, *n* = 9) on day 12 (i.e., at 5 days after SNI), along with the mean value. Two rats had overlapping values at approximately 29%. A cutoff of 70% ratio (i.e., an impairment of at least 30%) was used to determine the injured limb was impaired.

As seen in Figure 3A, the mean paw withdrawal threshold (PWT) of the injured limbs (18.7 ± 1.9 g) was significantly reduced (*p* < 0.0001) relative to the mean PWT of the non-injured ones (64.4 ± 6.2 g). All animals developed mechanical hypersensitivity, as defined by the PWT ratios for the injured limb being less than 70% of the non-injured limb. As seen in Figure 3 for all animals, the ratio of PWTs (injured: non-injured) limb was 31 ± 4%.

### Impedance stability over time

**FIGURE 4:**
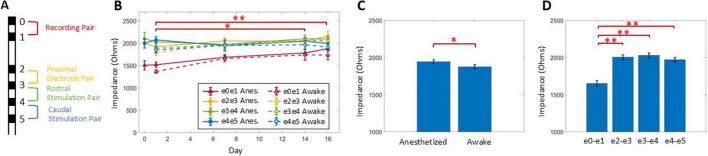
Impedances between neighboring contacts **(A)** were measured in awake and anesthetized states over time **(B)**. Impedances were significantly lower for awake conditions as compared to anesthetized **(C)**, for recording electrode pair e0/e1 compared to the other electrode pairs **(D)**, and for day 1 compared to day 14 and 15 (summarized in **B**). Statistical significance: *indicates *P* < 0.05, ^**^indicates *P* < 0.001.

Impedances between neighboring electrodes, with the configuration shown in Figure 4A, indicated no breakages occurred for any of the 9 animals over the course of the experiment. Comparing over time (Figure 4B), impedances collected on day 1 were significantly lower compared to day 14 and 16 (*P* < 0.002). Impedances were stable from day 7 onward, with no significant differences between day 7, 14, and 16. Mean impedances were significantly lower for awake conditions than for anesthetized conditions (*P* < 0.02) (Figure 4C) by 80 ± 31 Ohms. Impedances were also significantly lower for the recording (i.e., most rostral) electrode pair compared to the other electrode pairs (*P* < 0.001) by at least 319 ± 44 Ohms, when comparing e0/e1 impedances to e4/e5. No further significant differences were observed between the proximal electrode pair, the rostral stimulation pair, or the caudal stimulation pair (Figure 4D).

### Relationship between ECAPTs and vMTs

ECAPs were observed in all nine rats for all conditions tested for all 16 days. The confidence interval (CI) of ECAPTs was under 5 μA for 91% of growth curves and under 15 μA for 98% of growth curves analyzed by the algorithm (*n* = 640 growth curves total, after excluding 7 curves that were manually corrected).

**FIGURE 5:**
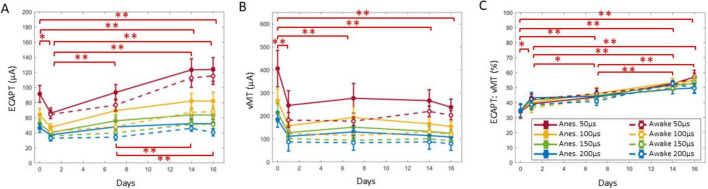
ECAPT **(A)** was calculated for each growth curve collected for each condition and was divided by the vMT **(B)** to create the ECAPT:vMT ratio **(C)**. A GLME model found day to be a significant factor for all three measures, and the summary statistics over time are shown here. Overall, the ECAPT decreased significantly from day 0 to 1, where it was the lowest compared to all other days. The ECAPT then significantly increased on day 7 (to a value similar to day 0). ECAPT continued to increase post SNI to day 14, but no further differences were observed after chronic SCS on day 16. Notably, vMTs **(B)** revealed that only the implantation day was significantly different, and all subsequent days did not have further differences. The ECAPT:vMT ratio significantly increased from day 0 to subsequent days until day 14 and 16, where no further changes to the ratio was observed after chronic SCS. Significant comparisons performed within these groups are summarized with asterisks, where *indicates *P* < 0.05 and ^**^indicates *P* < 0.001. Plots show mean ± standard error.

**FIGURE 6:**
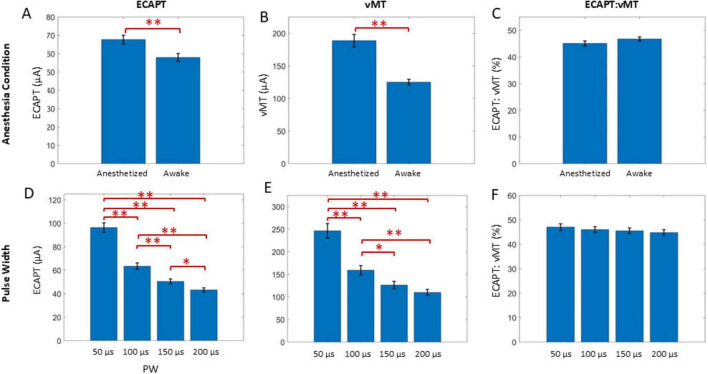
A GLME model also found the anesthesia state (top row) and PW (bottom row) to be significant factors for ECAPT **(A,D)** and vMT **(B,E)**, but not for ECAPT:vMT ratio **(C,F)**. Significant comparisons performed within these groups are summarized with asterisks, where *indicates *P* < 0.05 and ^**^indicates *P* < 0.001. Plots show mean ± standard error.

Across all animals, ECAPT significantly varied over time (Figure 5A), anesthesia state (Figure 6A) and PW (Figure 6D). Specifically, ECAPTs at day 0 were significantly greater than at day 1 (*P* < 0.004), and smaller than at day 14 or day 16 (*P* < 0.001). By day 7 ECAPTs had increased to be similar to those measured at day 0, and then continued to increase at day 14 and day 16 (*P* < 0.001), with no significant differences were observed before (day 14) and after application of SCS (day 16). Compared to recordings performed under anesthesia, awake ECAPTs were significantly lower (*P* < 0.001). As expected, ECAPTs decreased with increasing PW.

In terms of motor activation, vMTs were significantly higher on day 0 than at subsequent days (*P* < 0.001), with no further significant differences noted in the chronic recordings (Figure 5B). Similar to ECAPTs, vMTs were significantly lower in awake animals (*P* < 0.001) (Figure 6B) and with increasing PWs (Figure 6E).

To compare the relationship between the changes in neural and muscle activation, the ECAPT:vMT ratio was calculated and compared across recording conditions. Notably, there were significant differences detected over time (Figure 5C). The ECAPT: vMT ratio was lowest for day 0 at an average of 35 ± 2% (*n* = 72 growth curves across all conditions), and progressively increased to 40 ± 1%, 44 ± 1%, 51 ± 1%, and 54 ± 1%, on days 1, 7, 14, and 16 (*n* = 143–144 growth curves each), with differences across successive measurement being significant for all but the last two measurement (day 14 and 16). No significant differences were detected between anesthesia/awake state or PW (*P* > 0.05) (Figures 6C,F), indicating that these conditions had similar effects on neural and muscle activation.

### Slope of growth curves

**FIGURE 7:**
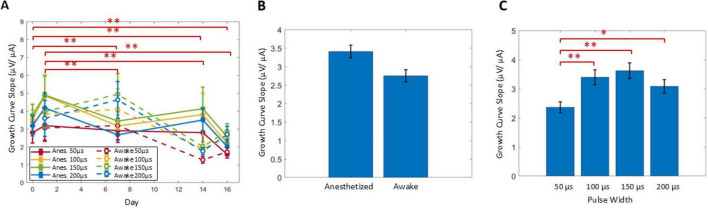
The slope of the growth curve was analyzed across days **(A)**, relative to anesthesia conditions **(B)** and among stimulus PWs **(C)**. Significant comparisons performed within these groups are summarized with asterisks, where *indicates *P* < 0.05 and ^**^indicates *P* < 0.001. Plots show mean ± standard error.

In addition to ECAPTs, the calculated slopes of the growth curves were also analyzed (Figure 7). Comparing over time (Figure 7A), slopes at day 0 and 1 were significantly different from the ones at day 7 and onward (*P* < 0.001). No significant differences were observed between slopes at days 7, 14, or 16, indicating no effect from SNI (day 7 vs. day 14) or SCS (day 14 vs. day 16), and similarly there were no significant differences detected with anesthesia state (Figure 7B). The slope was most shallow for the 50 μs PW (*P* < 0.01) compared to the longer PWs, with no further significant differences between the 100, 150, and 200 μs PWs (Figure 7C).

### Latency of ECAPs

**FIGURE 8:**
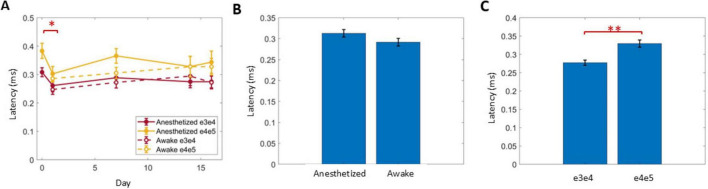
The latency of ECAPs just above 20 μV in response to 50 Hz stimuli with 50 μs PW was analyzed across days **(A)**, relative to anesthesia conditions **(B)** and two different stimulus locations **(C)**. Significant comparisons performed within these groups are summarized with asterisks, where *indicates *P* < 0.05 and ^**^indicates *P* < 0.001.

The latency of N1 was calculated for the first ECAP that exceeded 20 μV within a growth curve in response to stimulation at 50 μs PW (Figure 8). Latency significantly decreased from day 0 to 1 (*P* < 0.004), but no further differences were observed across days (Figure 8A) or with anesthesia condition (Figure 8B). The caudal stimulation pair e4/e5 had significantly longer latencies than the rostral stimulation pair e3/e4 by 53 ± 9 μs (P < 0.001). Averaging across all conditions recorded on days 1–16, the conduction velocity (CV) of ECAPs was 40 ± 1.4 m/s (*n* = 69) when stimulating with the caudal stimulation pair (e4/e5).

## Discussion

In this study, a common SNI-SCS model approach for neuropathic pain was used in anesthetized and awake rodents to explore changes in electrode impedances and both neural and muscular evoked activity over time. These recordings—with the animals serving as their own controls—enabled comparison of the effects of acute healing on day 1 post lead implantation, chronic healing on day 7, neural injury on day 14 (i.e., 7 days after SNI), and after continuous DTMP-SCS on day 16. After 5 days of SNI induction, all animals developed mechanical allodynia with an average of 70% decrease in paw withdrawal thresholds, similar to impairments previously observed with this model (Vallejo et al., 2020). The implications of these findings are discussed further below.

### Impedances and the electrode-tissue interface

In this work, impedances significantly increased from day 1 compared to day 14 and 16, though, no significant differences were noted from day 7 onward. Combined with the vMT and ECAPT changes noted from day 0 to 1, these observations suggest electrode-tissue interface maturation was ongoing at least through and beyond day 1 but had stabilized by day 7. Different physiological processes specific to the implanted lead location, geometry, and materials drive the maturation of the electrode-tissue interface in both the acute, post-surgical healing phase as well as the longer term, chronic healing phase. In the acute phase, a host of factors—primarily mediated by microglia—contribute to the inflammatory response at the electrode-tissue interface as blood and edema are cleared from the surgical site (Carnicer-Lombarte et al., 2021). After some days, healing transitions to the chronic phase, where astrocytes encapsulate the implant over the span of weeks to months and form a fibrotic layer. These processes can manifest in a change in the electrode impedance over time (Arle et al., 2014). While few prior reports of chronic SCS electrode impedance changes in rats exist, a study using titanium-nitride electrodes (versus the clinically relevant Pt-Ir electrodes used in this work) reported an initial impedance increase over the first 2 weeks post-implant followed by stable impedances thereafter for an additional 14 weeks (Garcia-Sandoval et al., 2018). Clinically, the impedances of SCS electrodes—when placed in the high thoracic spine to treat angina—were shown to decrease day 1 after implantation and stabilize within 1 month post-implant (Andersen, 1997). These impedance shifts inherently change the conductivity of the neural interface in a frequency-specific manner characterized by the Randles equivalent circuit (Randles, 1947), and are noted for both a variety of materials (Franks et al., 2005) and implant locations (both central and peripheral) (Straka et al., 2018); changes to the morphology and amplitude of the recorded ECAP may result if the signal amplifier is sensitive to these impedance shifts.

In addition to these biotic factors, the impedances verified that no breakages in the electrodes occurred throughout the experimental timeline. Two additional significant trends in impedances were evident that may be specific to this approach. First, the impedance was significantly higher while under anesthesia, which may be due to both changes in animal position and systemic effects of the isoflurane. Animals in awake states were upright in a tube and standing on four feet, while animals under anesthesia were prone with limbs extended. Isoflurane also results in peripheral vasodilation (Constantinides et al., 2011) and decreased serum ion concentrations (Goldberg et al., 1996), factors that can influence impedance measurements. Secondly, impedances were significantly lower for the recording electrode pair than the other electrode pairs. This may be due to the relative distance between contacts, since conductive surfaces—even if they are not electrically connected to other elements in the system—influence the distribution of electrical fields in an electrolytic medium (Single and Scott, 2018).

### Neural activation over time

Comparing to previous work, the ECAPT:vMT ratio of 35 ± 2% on the implantation day was similar to our previous study using similar parameters (Cedeño et al., 2023b). After day 1, the ECAPT: vMT ratio increased to 40 ± 1%, corresponding to vMTs being 2.5 times larger than ECAPTs—similar to values reported of 35% in acute studies (Dietz et al., 2022) and approximately 32–45% up to 2 days post implantation (Versantvoort et al., 2024). We further observed novel findings that the ratio increased over time to 54 ± 1% on day 16. Overall, these values are similar to prior reports suggesting paresthesia thresholds occur at 40–50% of MT for animals with neuropathic pain models (Shechter et al., 2013; Song et al., 2014; Cedeño et al., 2023a).

Novel results in this study allow the investigation of neural activation acutely and compare that to a longer term chronic healing as observed on day 7. Differences in ECAPTs, vMTs, the ECAPT:vMT ratio, and latency of ECAP were observed between day 0 and 1, indicative that day 0 data may have been influenced by the longer anesthesia period and surgical trauma. Thus, data collection on day 1 is critical in both demonstrating the plainly evident differences even within a small acute healing period and distinguishing these changes from longer term healing. By day 7, impedances and motor thresholds stabilized, suggesting that further changes in ECAPT and ECAPT:vMT ratio are not likely due to long-term healing effects. Similarly, the latency for a 20 μV ECAP had also stabilized for day 1 onward. The initial post-trauma (surgical or otherwise) latency prolongation likely represents a common finding across species for many types of evoked potentials. In guinea pigs, for instance, prolonged latencies are seen in auditory brainstem response post-acoustic trauma and in auditory nerve ECAPs post-surgical trauma; in both instances, the latency prolongation resolves after several days (Gourévitch et al., 2009; Ramekers et al., 2022).

The changes in ECAPT and ECAPT:vMT ratio over time contrast that of vMT, which exhibited no further significant changes after day 0. As such, the significant changes in ECAPT:vMT ratio both from day 1 and 7 onward are attributable solely to shifts in the ECAPTs versus shifts in the electrode impedance or vMT. One intriguing explanation for this observation is modulation of neural excitation in the dorsal columns by the SNI model. Notably, this effect stabilized, and no further differences were observed after the application of SCS, at least within the 2-day period of this study. The stability of this ratio after continuous SCS is relevant translationally as it suggests that DTMP does not alter the electrophysiology of the dorsal column Aβ fibers from which ECAPs originate. DTMP, which is shown to have significant improvement of mechanical hypersensitivity in rats compared to low-rate or high-rate stimulation alone, has been shown to modify the expression of glial genes toward a non-pain state (Vallejo et al., 2020), but the effect on neural excitation had not been studied previously. Moreover, this result indicated that dosing is consistent over time, given that SCS in translational models is typically dosed relative to vMT and not further adjusted after therapy begins. In contrast, changes in this ratio after SCS would have indicated that therapy would require adjustment in amplitude over time to achieve consistent dosing. The similarity in ECAPT:vMT ratio before and after continuous SCS agrees with previous findings of consistent sensory thresholds in similar rat SNI models after 3 days of either low or high frequency stimulation (Shechter et al., 2013).

### Effect of pulse width on neural activation

We confirmed the effect of PW from 50 to 200 μs on growth curves trends across time and neural activation metrics. As expected from the classic strength-duration curve relationship (Brunel and Van Rossum, 2007), an increase in PW decreased ECAPT and vMT as more charge was delivered to the tissue. Similar to previous work (Cedeño et al., 2023b; Versantvoort et al., 2024), the PW did not affect the ECAPT: vMT ratio up to 16 days after implantation. While ECAPs were observed for all PWs tested in this study, the stimulation artifact elicited by the longer PWs partially effaced the N1 feature within the ECAP, especially in response to the 200 μs PW. The artifact obstruction may have had a small effect on the slope of the growth curve, given that the 200 μs PW results were on average lower (but not significantly) than with the 100 or 150 μs PWs.

### Effects of anesthesia

In this study, the anesthetic agent (isoflurane) significantly increased impedances, ECAPTs, and vMTs, but had no effect on ECAPT: vMT ratios, the slope of the growth curve, or the latency of a 20 μV ECAP. The effect of isoflurane increasing motor thresholds has previously been described in rats approximately 1 day (Dietz et al., 2022) and 1 month (Yang et al., 2011) after implantation of an SCS lead. Overall, isoflurane has a depressive effect of muscle activity across a variety of preclinical models, where the anesthesia significantly reduces compound muscle action potentials (CMAPs) in amplitude in mice (Osuchowski et al., 2009) after 5 min of exposure. Similar results have been noted in cats (Yamada et al., 1995), Nubian goats (Andel et al., 2000) and humans (Rohde et al., 2003).

In addition to blocking motor responses, isoflurane has been shown to decrease neural excitability of neurons in the dorsal horn (Yang et al., 2021) by binding to GABA and glycine receptors (Grasshoff and Antkowiak, 2006). In ovines, ECAPT and growth curve slopes were lower for awake conditions than under isoflurane when recorded on the same day (Goodman et al., 2020). In contrast to our results where ECAPTs were lower in awake states, Dietz et al found that ECAPTs were comparable between conditions (Dietz et al., 2022). Notably, in that study, unanesthetized recordings were collected at least 4 h after anesthetized condition, which was taken immediately after implantation. In addition, while we did not observe difference in latencies (and thus conduction velocities) in spinal ECAPTs between anesthetic states, previous nerve conduction studies in mice found that isoflurane decreases nerve conduction after 5 min of exposure compared to awake conditions (Osuchowski et al., 2009), though the impact on CV is less in comparison to other anesthetics (Oh et al., 2010). Such differences in latencies or CV may require leads with greater site separation than the 1-mm inter-electrode spacing used here to detect differences in future research.

### Latencies and conduction velocity

The conduction velocities recorded here averaged at 40 ± 1.4 m/s, similar to orthodromically recorded ECAPs in previous studies (Dietz et al., 2022). These results suggest that ECAPs from large, myelinated fibers were acquired. No differences in latencies after day 1 or between anesthesia condition was observed, contrasting a prior report of antidromic CVs being significantly slower in SNI animals as compared to sham (Versantvoort et al., 2024). These differences may be due to inter-animal differences, especially as only 4–5 animals were allocated per group in the prior study, or due to the limited sampling resolution of 33 μs (as compared to the resolution of 25 μs in this study). In contrast, this study used only the latencies from one ECAP amplitude for consistency (i.e., just exceeding 20 μV) to assess latency differences across different animals and conditions.

### Clinical translation

These results highlight acute changes in the neural interface with translational impacts. Accordingly, caution is inculcated for the clinician using SCS systems that rely on stability of the electrode-tissue interface—such as ECAP-controlled, SCS systems that deliver closed-loop paresthesia at a specific ECAP amplitude (Russo et al., 2018)—as this interface is still maturing perioperatively. Establishing parameters for closed-loop therapy while these changes are underway may result in poor dose control or un-optimized therapy for some patients. For example, as the ratio between neural and motor threshold increases, this would likely be translated to differences in neural and sensory thresholds. Additionally, systems that use a template for ECAP identification may need adjustments as the morphology of the ECAP changes with N1 latency decreases. In humans, these factors may be acutely compounded, where some contacts have high impedances immediately post implantation due to blood or air bubbles that are then resolved in subsequent days. Importantly, the stability in ECAPT as well as ECAPT:vMT after 2 days of continuous DTMP in this study suggests that neural excitability does not change further—a clinically meaningful outcome, as further modulation would likely indicate therapy reprograming. Similar to work demonstrating how small changes in distance between the lead contacts and the spinal cord impacts neural activation (Brucker-Hahn et al., 2023), future computational modeling work could illuminate how differences in the electrode-tissue interface impacts the activation of Aβ fibers.

### Study limitations

The study was structured to mimic previous SNI studies, where each animal served as its own control for pre-SNI (days 1–7), post SNI (day 14), and post SCS (day 16). The limitation in this approach is that the stability of ECAPs from days 14 + was not evaluated without the influence of SNI. However, a recent study compared animals with and without SNI injury for 2 days post SCS and did not find significant differences in the MT:ECAPT ratios between sham and SNI animals (Versantvoort et al., 2024). Moreover, the limited sample size of 9 animals, while similar to previous work and is sufficient to validate the SNI model, limits some statistical comparisons in which experimental variance was larger (such as latency in awake verses anesthetized animals). Another limitation in this study was that the effect of continuous SCS was only taken over 2 days, which was chosen to follow previous studies investigating the behavioral effects and mechanism of action of DTMP (Cedeño et al., 2020, 2023a; Vallejo et al., 2020). Future studies should explore longer-term effects of DTMP-SCS on ECAPs. Additional methodological constraints include that PWTs were only measured 5 days post-SNI to validate that the animals had developed the model, and did not measure them pre- and post-SCS. This approach, which reduced the number of experimental interventions, used previous literature (Decosterd and Woolf, 2000) and our own experience (Tilley et al., 2015; Vallejo et al., 2020) to validate the sustainability of the model. Additionally, there were some practical limitations of the recordings, including placing the reference needle and restraining the animal in a tube to minimize movement artifacts. However, the impact on elicited neural activity is likely minimal given the lack of significant differences detected between anesthetized states for the ECAPT: vMT ratios, the slope of the growth curve, or the latency of a 20 μV ECAP. Finally, it was not possible to collect x-ray images for all animals in the study, although for the first 5 animals lead placement was confirmed to be within one vertebral body of the target T13, suggesting that this electrode array configuration and surgical technique enabled consistent placement.

## Conclusion

This study provides novel evidence demonstrating the feasibility of recording spinal ECAPs with clinically relevant electrode configurations in rats over the span of weeks. Over time, the relationship between ECAPT and MTs varies both due to healing as well as nerve injury, and this provides crucial insights into SCS dosing for translational research. This approach could be leveraged for a variety of future studies searching to elucidate mechanisms of action and neurophysiological changes underpinning SCS therapies.

## Data Availability

The datasets presented in this article are not readily available because the datasets are the property of Medtronic plc. Requests to access the datasets should be directed to david.a.dinsmoor@medtronic.com.
