# Supplementary material for: Evolution of spinal evoked compound action potential thresholds, visual motor thresholds, and impedances in a rodent spared nerve injury model
Source: Front Neurosci. 2025 Jun 30;19:1577059. doi: 10.3389/fnins.2025.1577059 (PMC12256503; doi:10.3389/fnins.2025.1577059)
Supplement: Supplementary file 1 [file Table_1.docx]

# Supplementary Material

For each response variable, a GLME model was fit in MATLAB using the function *fitglme,* where the random effect was the rat; this function does not allow for multiple response variable in a model. Fixed effects for ECAPTs, vMTs, ECAPT:vMT ratios, and growth curve slope included the anesthesia state, the PW, and the day post implantation. Initial analyses of using Impedance as a fixed effect for ECAPT found a detrimental impact as evidenced by a higher AIC and BIC, leading us to exclude it from further consideration. Fixed effects for latency and impedance included the anesthesia state, the recording configuration, and the day post implantation. All fixed and random effects were categorical variables for subsequent comparison tests. Summary variance statistics for each model are shown in Table 1. The Random Effect Variance was calculated with the *covarianceParameters* function. To calculate the ICC, the Random Effect Variance was divided by the sum of the Random Effect Variance and the Residual Variance (found by dividing the SSE by the DFE).

**Table 1:** A summary of the variance statistics for each GLME model

| Response Variable | Adjusted R2 | Random Effect Variance | SSE | SST | SSR | DFE | ICC |
| --- | --- | --- | --- | --- | --- | --- | --- |
| ECAPT | 0.70 | 611.2 | 3.24E+05 | 1.09E+06 | 7.62E+05 | 638 | 0.55 |
| vMT | 0.54 | 7340.3 | 6.40E+06 | 1.41E+07 | 7.69E+06 | 638 | 0.42 |
| ECAPT:vMT | 0.42 | 57.9 | 8.77E+04 | 1.53E+05 | 6.51E+04 | 638 | 0.30 |
| GCslope | 0.47 | 1.8 | 3.14E+03 | 5.99E+03 | 2.85E+03 | 632 | 0.27 |
| Latency | 0.44 | 0.002 | 0.512 | 0.946 | 0.434 | 150 | 0.34 |
| Impedance | 0.36 | 1.3E+04 | 2.17E+07 | 3.50E+07 | 1.32E+07 | 315 | 0.16 |
